# Glucose and Fatty Acids Synergize to Promote B-Cell Apoptosis through Activation of Glycogen Synthase Kinase 3β Independent of JNK Activation

**DOI:** 10.1371/journal.pone.0018146

**Published:** 2011-04-26

**Authors:** Katsuya Tanabe, Yang Liu, Syed D. Hasan, Sara C. Martinez, Corentin Cras-Méneur, Cris M. Welling, Ernesto Bernal-Mizrachi, Yukio Tanizawa, Christopher J. Rhodes, Erik Zmuda, Tsonwin Hai, Nada A. Abumrad, M. Alan Permutt

**Affiliations:** 1 Division of Endocrinology, Metabolism, and Lipid Research, Washington University School of Medicine, St. Louis, Missouri, United States of America; 2 Division of Nutritional Science, Department of Medicine, Washington University School of Medicine, St. Louis, Missouri, United States of America; 3 Division of Endocrinology, Metabolism, Hematological Sciences and Therapeutics Department of Bio-Signal Analysis, Yamaguchi University Graduate School of Medicine, Ube, Yamaguchi, Japan; 4 Department of Medicine, Kovler Diabetes Center, University of Chicago, Chicago, Illinois, United States of America; 5 Department of Molecular and Cellular Biochemistry, Center for Molecular Neurobiology, Ohio State University, Columbus, Ohio, United States of America; University of Bremen, Germany

## Abstract

**Background:**

The combination of elevated glucose and free-fatty acids (FFA), prevalent in diabetes, has been suggested to be a major contributor to pancreatic β-cell death. This study examines the synergistic effects of glucose and FFA on β-cell apoptosis and the molecular mechanisms involved. Mouse insulinoma cells and primary islets were treated with palmitate at increasing glucose and effects on apoptosis, endoplasmic reticulum (ER) stress and insulin receptor substrate (IRS) signaling were examined.

**Principal Findings:**

Increasing glucose (5–25 mM) with palmitate (400 µM) had synergistic effects on apoptosis. Jun NH2-terminal kinase (JNK) activation peaked at the lowest glucose concentration, in contrast to a progressive reduction in IRS2 protein and impairment of insulin receptor substrate signaling. A synergistic effect was observed on activation of ER stress markers, along with recruitment of SREBP1 to the nucleus. These findings were confirmed in primary islets. The above effects associated with an increase in glycogen synthase kinase 3β (Gsk3β) activity and were reversed along with apoptosis by an adenovirus expressing a kinase dead Gsk3β.

**Conclusions/Significance:**

Glucose in the presence of FFA results in synergistic effects on ER stress, impaired insulin receptor substrate signaling and Gsk3β activation. The data support the importance of controlling both hyperglycemia and hyperlipidemia in the management of Type 2 diabetes, and identify pancreatic islet β-cell Gsk3β as a potential therapeutic target.

## Introduction

The natural history of Type 2 diabetes mellitus (T2D) includes a progressive decline in β-cell function associated with peripheral insulin resistance. The β-cell dysfunction has been attributed in part to loss of β-cell mass via apoptosis [1] with inadequate insulin secretion leading to hyperglycemia and other diabetes symptoms [2]. Insulin resistance is at the core of obesity associated diabetes and is thought to reflect impaired insulin signaling due to chronically increased levels of free fatty acids (FFA). High FFA are also implicated in the reduction in β-cell mass that has been referred to as lipotoxicity. The combination of elevated glucose and FFA, or “glucolipotoxicity” that is prevalent in T2D has been suggested to be a major contributor to β-cell death [3], [4], [5], [6].

The search for molecular mechanisms for glucose potentiation of FFA- induced β-cell dysfunction has been the subject of several recent studies (see [7] for review). One area of investigation has focused on the insulin receptor substrate-PI3K-Akt signaling pathway. The first study showing that the FFA oleate impaired insulin signaling in insulinoma cells demonstrated that the cells were protected from FFA-induced apoptosis by expressing a constitutively active Akt [8]. Several biochemical and genetic studies subsequently showed that saturated FFA could promote ER stress in insulinoma cells and in primary rodent and human islets [9], [10], [11], [12]. More recently, it was shown that high glucose potentiated FFA induced apoptosis by enhancing ER stress [13]. ER stress in insulinoma cells was shown to impair insulin signaling through activation of ATF3, an ER stress response protein that was implicated in suppression of IRS2 expression [14]. ATF3 is another stress inducible gene that is activated in different tissues by a variety of stresses [15].

How glucose potentiates FFA induced ER stress, reduced insulin receptor substrate signaling, and apoptosis is incompletely understood. Our recent study showed that there was a dose-dependent effect of FFA in the presence of high glucose on apoptosis in insulinoma cells and primary islets [16] that was associated with JNK activation, ER stress, and reduced insulin signaling. In the current study, we found a dose-dependent effect of glucose in the presence of palmitate on cell death that appeared to be over and above JNK activation. We observed glucose dose-dependent synergistic effects on palmitate inhibition of receptor substrate signaling and activation of Gsk3β. Co-treatment with an adenovirus expressing a kinase dead Gsk3β significantly protected β-cells from cell death. Our data support importance of Gsk3β in the synergistic effects of glucose and FFA.

## Materials and Methods

### Cell Culture

Mouse insulinoma cell line MIN6 (passage 24–32) were grown in monolayer cultures as described previously [17] in Dulbecco's modified Eagle's medium (Sigma Aldrich) supplemented with 15% fetal bovine serum, 50 mmol/L β-mercaptoethanol at 37°C in a humidified (5% CO_2_, 95% air) atmosphere. Rat insulinoma INS-r3 cells were grown as previously described [18]. The palmitic acid (palmitate), formalin, propidium iodide, IL-1b, tunicamycin and TNFα were purchased from Sigma (Saint Louis, MO). Tauroursodeoxycholic Acid Sodium Salt (TUDCA) was purchased from CALBIOCEM (Darmstadt, Germany).

### Fatty acids (FFA) Treatment of MIN6 Cells and Islets

The complete protocol was previously described [16]. Briefly a 20 mM solution of the FFA in 0.01 M NaOH was incubated at 70°C for 30 minutes. Then, 330 µL of 30% BSA and 400 µL of the free FFA/NaOH mixture was mixed together and filter sterilized with 20 mL of either the DMEM or RPMI media. The approximate molar ratio of FFA∶BSA is 6∶1 with 400 µM palmitate. The addition of BSA or a FFA∶BSA mixture has not been shown to affect the pH of the media.

### Propidium iodide/Cell Death Assay

MIN6 cells were grown on glass cover slips within the wells of a 6-well plate and incubated with either BSA alone or 400 µM both FFAs complexed with BSA for 24 hours as previously described [16]. After treating cells for 24 hours with various treatments, the cells were incubated with 10 µg/ml (1 to 1000 dilution) Propidium Iodide (PI) and 20 ug DAPI added directly to the media at 37°C, 5% CO2 for 1 hour. The medium was then removed by aspiration, and the cells were washed once with PBS and then fixed by incubation with 3.7% formaldehyde for 15 min at room temperature. After fixation, the MIN6 cells were mounted with anti-fading gel solution including DAPI (Biomeda Corporation, Foster City, CA) on to glass slides. Each condition reported represents over 3000 cells counted by randomized field selection. The percentage of cell-death is reported as the number of PI stained nuclei over the total number of nuclei stained by DAPI as quantitated by Image J software 1.37 [19].

### Western blot analysis

MIN6 were washed twice in ice-cold phosphate-buffered saline and were lysed in ice-cold cell lysis buffer consisting of 50 mM HEPES (pH 7.5), 1% (v/v) Nonidet P-40, 2 mM activated sodium orthovanadate, 100 mM sodium fluoride, 10 mM sodium pyrophosphate, 4 mM EDTA, 1 mM phenylmethylsulfonyl fluoride, 1 µg/mL leupeptin, and 1 µg/mL aprotinin, then passed through syringe with a 21 gauge needle 10 times while INS-r3 cells were sonicated (Misonix, Farmingdale, NY) and particulate material from both cell lines were removed by centrifugation (10,000× g; 10 min; 4°C). The supernatants were collected. Protein concentrations were determined using the Bio-Rad protein assay (Bio-Rad, Hercules, CA).

The extracts (20 µg of total protein) were resolved on 7.5% or 4–15% gradient polyacrylamide gels and were blotted onto a nitrocellulose membrane (Bio-Rad, CA), and incubated overnight at 4°C in Tris-buffered saline containing a 1∶1000–5000 dilution of antibody as listed below. The membrane was then incubated at 4°C for 60 min in Tris-buffered saline with a 1∶2000 dilution of anti-rabbit IgG or anti-mouse IgG horseradish peroxidase-conjugated secondary antibody (Cell Signaling Technology). Antibodies used were anti-total Akt, anti-phospho-Akt (S473), anti-cleaved Caspase3, anti-phospho-PERK (980Thr), anti-phospho-eIF2α(51Ser), anti-total JNK1/2, anti-phospho-JNK, anti-phospho-AMPK, total AMPK, anti-phospho-ACC, anti-total ACC from Cell Signaling Technology (Beverley, MA), anti-SREBP1 from Neo Markers (Fremont, CA), anti-IRS1, anti-IRS2 from Upstate (Billerica, MA), anti-ATF3, anti-Insig1, anti-Lamin from Santa Cruz (Santa Cruz, CA) and anti-α-Tubulin and from Sigma (Saint Louis, MO).

Immune complexes were revealed using ECL Advance Western Blot Detection kit (Amersham Plc, Buckinghamshire UK) and the images were acquired using a FluoroChem 8800 digital camera acquisition system (Alpha Innotech, San Leandro, CA, USA). Band intensities in the blots were later quantified using ImageJ 1.38× [19] and α-Tubulin or β-Actin bands were used to adjust for loading differences.

### Nuclear and cytoplasmic fractions from MIN6

MIN6 cells were cultured in 60-mm diameter culture dishes until 80% confluency. For isolation of nuclear extracts, the cells were then collected into microtubes, centrifuged for 20 s in a microcentrifuge, and resuspended in 200 µl of 10.0 mM Hepes, pH 7.9, containing 10.0 mM KCl, 1.5 mM MgCl_2_, and 0.5 mM dithiothreitol. After incubation at 4°C for 15 min, the cells were lysed by passing 10 times through a 21-gauge needle. Next, the cells were centrifuged for 20s in a microcentrifuge, and the supernatant (cytoplasmic fraction) was removed and frozen in small aliquots. The pellet, which contained the nuclei, was resuspended in 150 µl of 20 mM Hepes, pH 7.9, containing 20% v/v glycerol, 0.1 M KCl, 0.2 mM EDTA, 0.5 mM dithiothreitol, and 0.5 mM phenylmethanesulfonyl fluoride and then stirred at 4°C for 30 min. The nuclear extracts were then centrifuged for 20 min at 4°C in a microcentrifuge. The supernatant was collected, aliquoted into small volumes, and stored at −80°C.

### Islet isolation and culture

Islets from 12 weeks of age C57BL/6 male mice were isolated by ductal collagenase distension/digestion of the pancreas [20] followed by filtering and washing through a 70 mm Nylon cell strainer (BD Biosciences, San Jose, CA). Isolated islets were then maintained in RPMI medium containing 11 mM glucose, 10% FBS, 200 units/ml penicillin, and 200/ml streptomycin in humidified 5% CO2, 95% air at 37C. The palmitate treatments were carried out 15 hours after isolation. Adenovirus infections were initiated immediately following isolation at 500 multiplicity of infection (MOI). Infections were incubated for 15 hours and residual virus was removed prior to palmitate treatment. All procedures were performed in accordance with Washington University's Animal Studies Committee. The Principles of laboratory animal care (NIH publication no. 85–23, revised 1985; http://grants1.nih.gov/grants/olaw/references/phspol.htm) were followed.

### Loss-of-function of ATF3 with shATF3

INS-r3 cells were seeded 24 hours prior to infection to achieve 70 percent confluence at time of infection. Control and ATF3 shRNA adenovirus constructs described previously [14] were incubated with the cells at an MOI of 30 for 4 hours in normal culture media. Palmitate and cytokine treatments were initiated 24 hours following removal of virus.

### Inhibition of Gsk3β expression with a kinase dead adenovirus

Adenovirus expressing a catalytic inactive mutant of the human Gsk3β (Adv-Gsk3βKM) was prepared as previously described [21]. Control adenovirus-green fluorescent protein (AdV-GFP) was a gift from D. Kelly (Washington University, St. Louis, MO). Infection of the MIN6 cells was carried out at the indicated multiplicity of infection (MOI) for one hour in serum-free media. The MIN6 were then washed in PBS, maintained in the DMEM/15% FBS media, and then experiments were carried out 24 hours after infection.

### Statistical analysis

The presented data were analyzed from at least 3 independent experiments and are shown as means ±S.E.M. The significance of the variations was analyzed using either a one- or two-way ANOVA with Bonferroni corrections with a significance level of 0.05 (95% confidence intervals).

## Results

### Glucose and palmitate synergize to induce apoptosis

**Figure 1:**
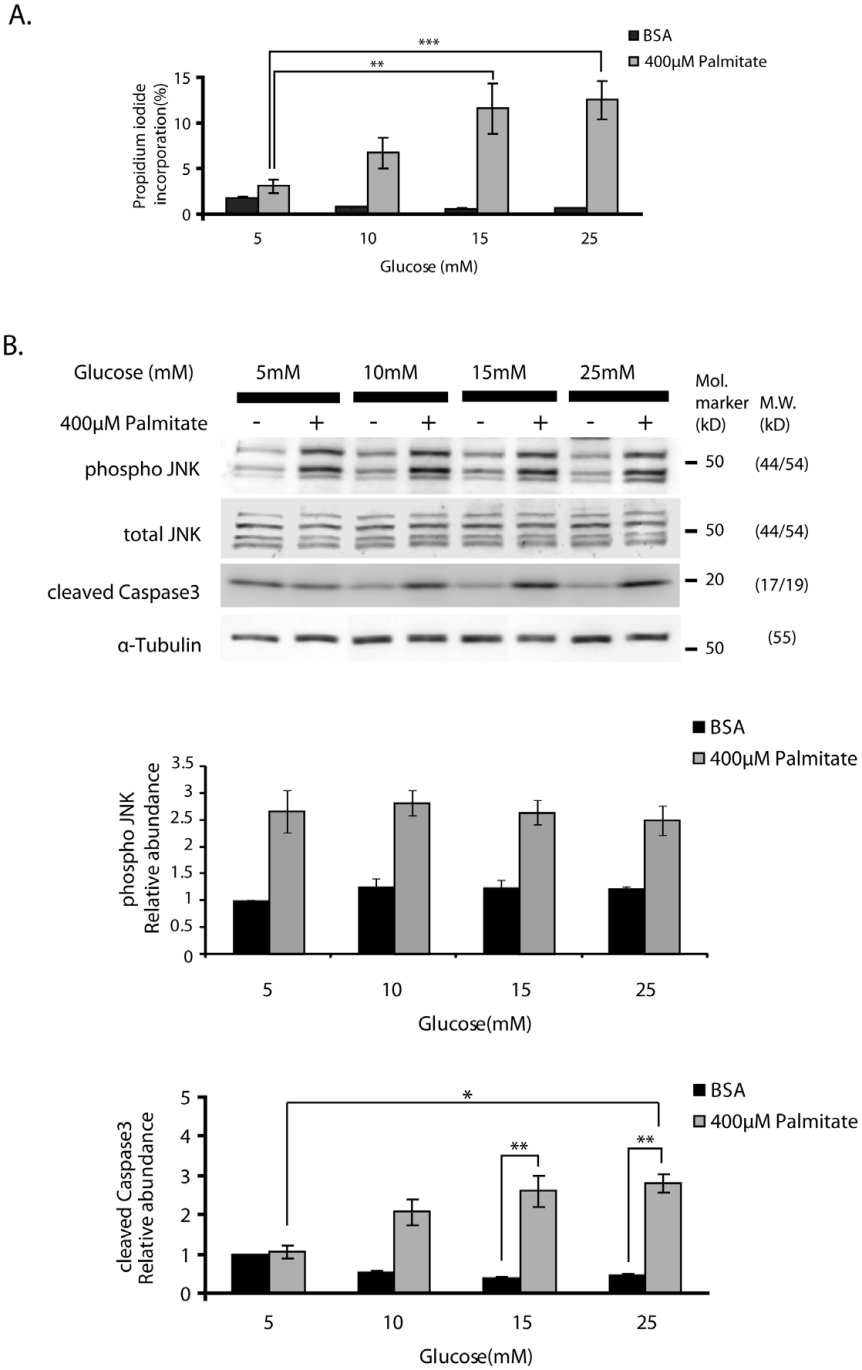
Synergistic effects of glucose and palmitate on cell death but not JNK activation in MIN6 cells. MIN6 cells were treated with either control 0.5% BSA or 400 µM palmitate+0.5% BSA at a concentration of 5, 10, 15, 25 mM glucose for 24-h. (**A**) The percentage of cell death was then assessed by adding propidium iodide for the last hour of incubation as described under Methods. The bar graph depicts the averages of the data obtained from five individual experiments, and data are expressed as means ±S.E.M. ** p<0.01, *** p<0.001; (**B**) The cell lysates were subjected to Western blot analysis using anti-cleaved Caspase3, anti-phospho-JNK, anti-total JNK and anti-α-Tubulin antibodies. Protein level of phospho-JNK was normalized over total JNK. Cleaved Caspase3 levels were normalized over α-Tubulin. The representative result of three individual experiments is shown. The data obtained from three individual experiments are expressed as means ± S.E.M. * p<0.05, ** p<0.01.

Our previous study had shown a dose-dependent effect of FFA, both palmitate (50–400 µM) and oleate (50–400 µM), on ER stress and apoptosis in glucose-responsive insulinoma (MIN6) cells [16]. In the current study the dose-dependent effect of glucose (5–25 mM) was examined at 400 µM palmitate. As shown in Figure 1A, increasing glucose concentration had a clear synergistic effect on cell death characterized by propidium iodide incorporation normalized to DAPI staining. While 400 µM palmitate resulted in about 3% propidium iodide incorporation at 5 mM glucose, this was increased more than threefold when the glucose concentration was raised to 15 mM and 25 mM. While there was no effect of palmitate on cleaved Caspase3 at 5 mM glucose, consistent with the synergism observed on propidium iodide staining, glucose and palmitate also synergized on activation of the pro-apoptotic marker cleaved Caspase3 comparing that at 5 mM glucose/palmitate vs. 25 mM glucose/palmitate, p<0.05 (Figure 1B).

We next explored the underlying mechanisms for the glucose potentiation. FFA treatment has been shown to induce JNK activation that can contribute to FFA-induced apoptosis; we determined the effects of altering glucose concentrations on JNK activation. Interestingly, JNK activation by FFA was maximal at the lowest glucose concentration and did not increase further as glucose was increased (Figure 1B). Maximal FFA activation of JNK at 5 mM glucose with apparent increasing activation of cleaved Caspase3 with increasing glucose concentration suggested that further enhancement of JNK by glucose could not explain the synergistic effects on cell death.

### Glucose and palmitate synergize to reduce insulin signaling associated with a decrease in IRS2 protein

**Figure 2:**
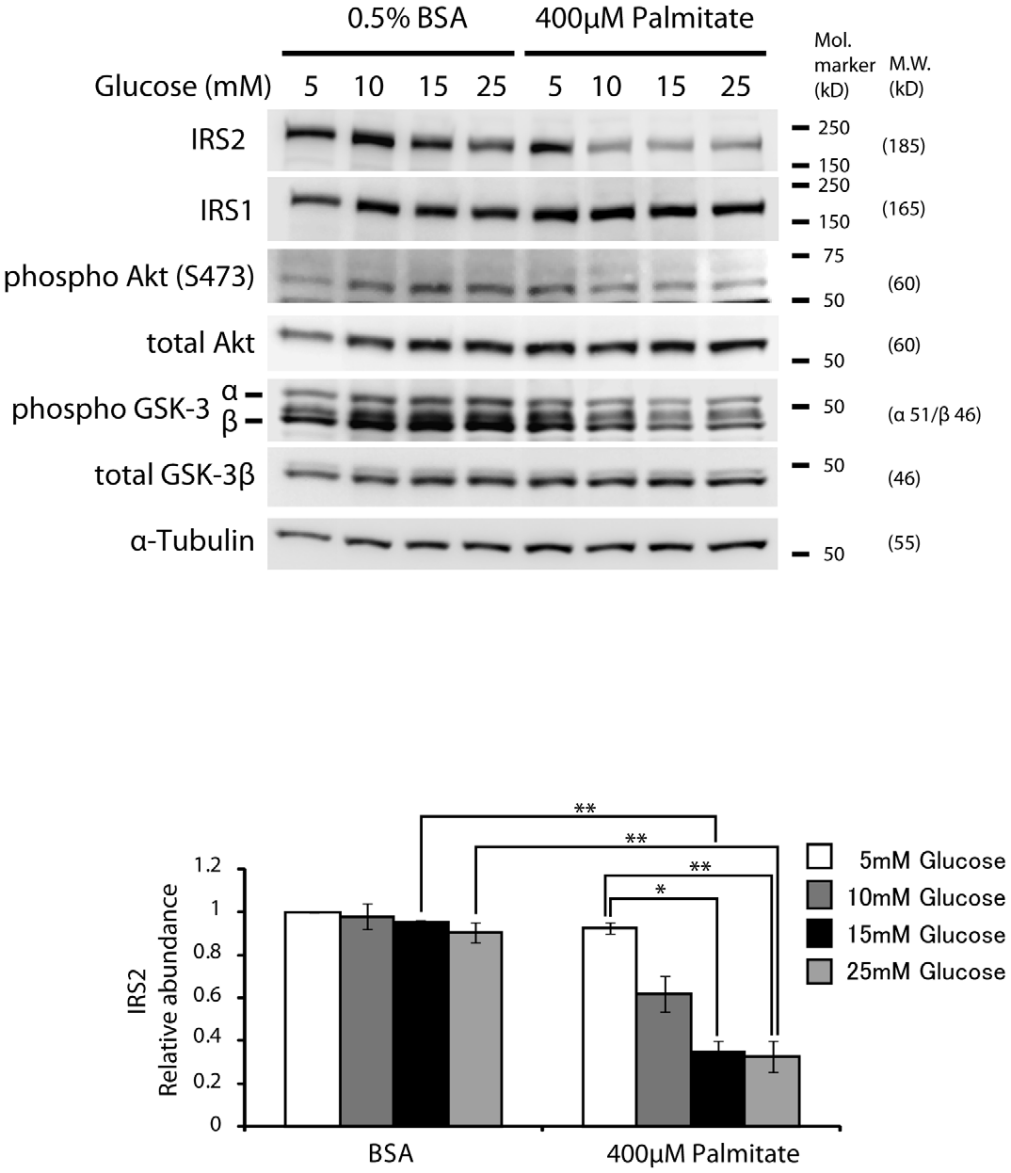
Glucose and palmitate potentiate to reduce insulin signaling. MIN6 cells were treated with either control 0.5% BSA (four lanes on left) or 400 µM palmitate+0.5% BSA (four lanes on right) at a concentration of 5, 10, 15, 25 mM glucose for 24-h. Total cell lysates were obtained and were subjected to Western blot analysis with antibodies to the indicated proteins. Protein level of IRS2 was normalized over α-Tubulin. The representative results of three individual experiments are shown. The results for IRS2 are graphically illustrated, data are expressed as means ±S.E.M. *p<0.05, **p<0.01.

Previous studies have shown that glucose treatment of insulinoma cells results in activation of the insulin receptor substrate-PI3-kinase-Akt pathway that serves to protect against β-cell death. In contrast, FFA treatment of insulinoma cells inhibits this pathway [8], [16]. We examined whether the synergistic effect of glucose on FFA-induced apoptosis could be related to inhibition of this signaling pathway. Glucose alone (5–25 mM), as shown in the first four lanes of Figure 2, increased phospho-Akt (S473) and phospho-Gsk3 as expected, with no apparent change in IRS1 or IRS2 proteins, confirming previous observations [8], [22]. However as glucose was raised in the presence of palmitate (Figure 2, lanes 5–8) a dose-dependent decrease in phospho-Akt and phospho-Gsk3β were observed and accompanied by a parallel decrease in IRS2, while IRS1 levels did not appear to change. This synergistic effect of increasing glucose in the presence of FFA on inhibition of IRS2 and PI-3 kinase-Akt signaling is a novel finding that might explain the synergistic effect on β-cell survival [23].

### The effect of glucose and FFA is accompanied by a synergistic effect on ER stress that is reduced by addition of a chemical chaperone

**Figure 3:**
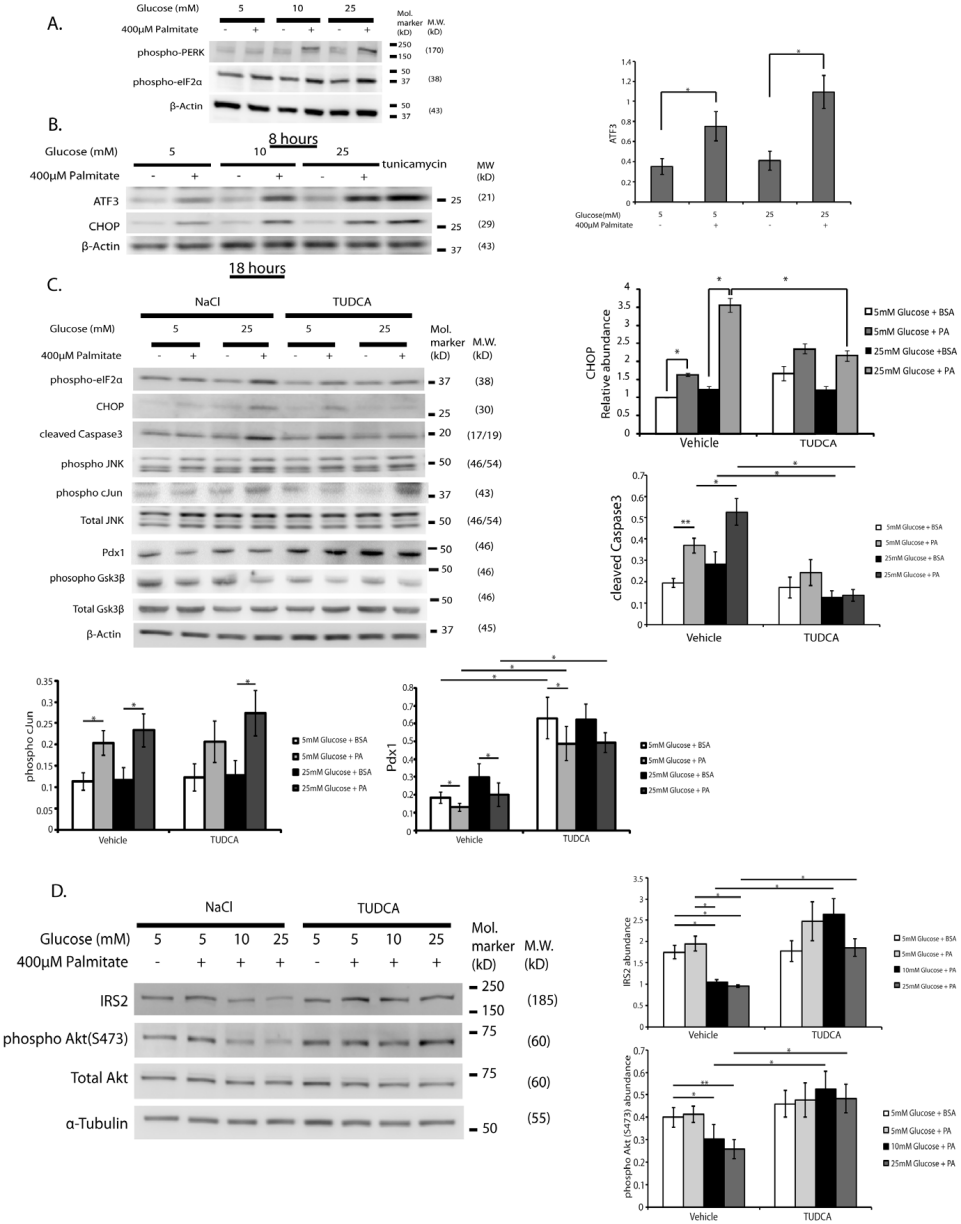
The synergistic effects of glucose and palmitate on ER stress and reduction of insulin signaling is attenuated by addition of a chemical chaperon. (**A**) MIN6 cells were treated with either control 0.5% BSA or 400 µM palmitate+0.5% BSA at a concentration of 5, 10, 25 mM glucose for 8 hours. Total cell lysates were extracted at indicated time points and were subjected to Western blot analysis using anti-phospho-PERK (980Thr), anti-phopsho-eIF2α (51Ser), and (**B**) MIN6 cells were treated with either control 0.5% BSA or 400 µM palmitate+0.5% BSA at a concentration of 5, 10, 25 mM glucose for 18 hours and blotted with anti-ATF3 and anti-CHOP antibodies. β-Actin was detected for loading control. Tunicamycin treatment was control for ER stress. (**C**) Cells were treated with either 500 µg/ml NaCl (ionic control) or 500 µg/ml TUDCA 15-h prior to beginning of palmitate treatment and then were co-treated with either 0.5% BSA or 400 µM palmitate+0.5% BSA with either 5 mM or 25 mM glucose and NaCl or TUDCA for 24 h. Total cell lysates were subjected to Western blot analysis with antibodies to the indicated proteins. Densitometry of total CHOP and cleaved Caspase3 and Pdx1 were measured and normalized over α-Tubulin, respectively. Densitometry of phospho-cJun was measured and normalized over total JNK. The representative results of three individual experiments are shown. The effects on CHOP, cleaved Caspase3 and phospho-cJun and Pdx1 protein are graphically illustrated. *p<0.05. (**D**) Cells were treated with either 500 µg/ml NaCl (ionic control) or 500 µg/ml TUDCA 15-h prior to beginning of palmitate treatment and then were co-treated with either 0.5% BSA or 400 µM palmitate+0.5% BSA with either 5 mM, 10 mM or 25 mM glucose and NaCl or TUDCA for 24-h. Total cell lysates were subjected to Western blot analysis with antibodies to the indicated proteins. Densitometry of total IRS2 was measured and normalized over α-Tubulin and densitometry of phospho-Akt was measured and normalized over total Akt. The representative results of three individual experiments are shown. The effects on IRS2 protein are graphically illustrated, *p<0.05, **p<0.01.

FFA impair insulin signaling in β-cells in part via activation of ER stress [16], [24]. Bachar et. al. [13] recently showed in insulinoma cells that palmitate at 22.2 mM glucose vs. 3.3 mM glucose increased activation of JNK, CHOP and the ER stress enzyme phospho-PERK. We examined the effects of increasing glucose from 5 mM to 25 mM at 8 and 18 hours of treatment to observe the different time course of development of ER stress markers. An enhancement of the ER stress markers phospho-PERK, phospho-eIF2α, CHOP, and ATF3 (Figure 3A and 3B) was observed, confirming and extending the results of Bachar et. al. [13]. The observation that glucose appeared to synergize with palmitate to increase ATF3 protein expression, although this was not statistically significant, was consistent with the previous finding by Hartman et. al. demonstrating that high glucose and FFA together increased ATF3 mRNA expression, and that this was associated with increased apoptosis of insulinoma cells [25]. These glucose-induced changes in the presence of palmitate on ER stress markers were again noted in the absence of phospho-c-Jun (Figure 3C).

We next examined whether reducing ER stress using TUDCA, a chemical chaperone that enhances ER functional capacity [26], can reverse the synergistic effects of glucose and FFA on insulin receptor substrate signaling. As shown in Figure 3C, the effects of 24 hour treatment with 5 mM or 25 mM glucose in the presence and absence of palmitate (400 µM) on activation of JNK and other markers of ER stress are shown in the control condition with NaCl (first 4 lanes). The results of protein expression under the same conditions but in the presence of TUDCA are shown in the last 4 lanes of Figure 3C. JNK activation, which again was not augmented by the combination of high glucose and palmitate, was little affected by TUDCA treatment. In contrast the other ER stress markers phospho-eIF2α, CHOP and cleaved Caspase 3 were attenuated by co-treatment with TUDCA. Addition of TUDCA also increased Pdx1 (Figure 3C). The increase in phospho-Akt protein levels, an inhibitor of Gsk3 activity, and IRS2 protein levels were also observed with TUDCA treatment (Figure 3D). Together the results suggest that glucose potentiation of FFA induced apoptosis involves activation of ER stress with resultant inhibition of insulin signaling in a manner independent of further JNK activation.

### Glucose and palmitate synergistically activate SREBP1

**Figure 4:**
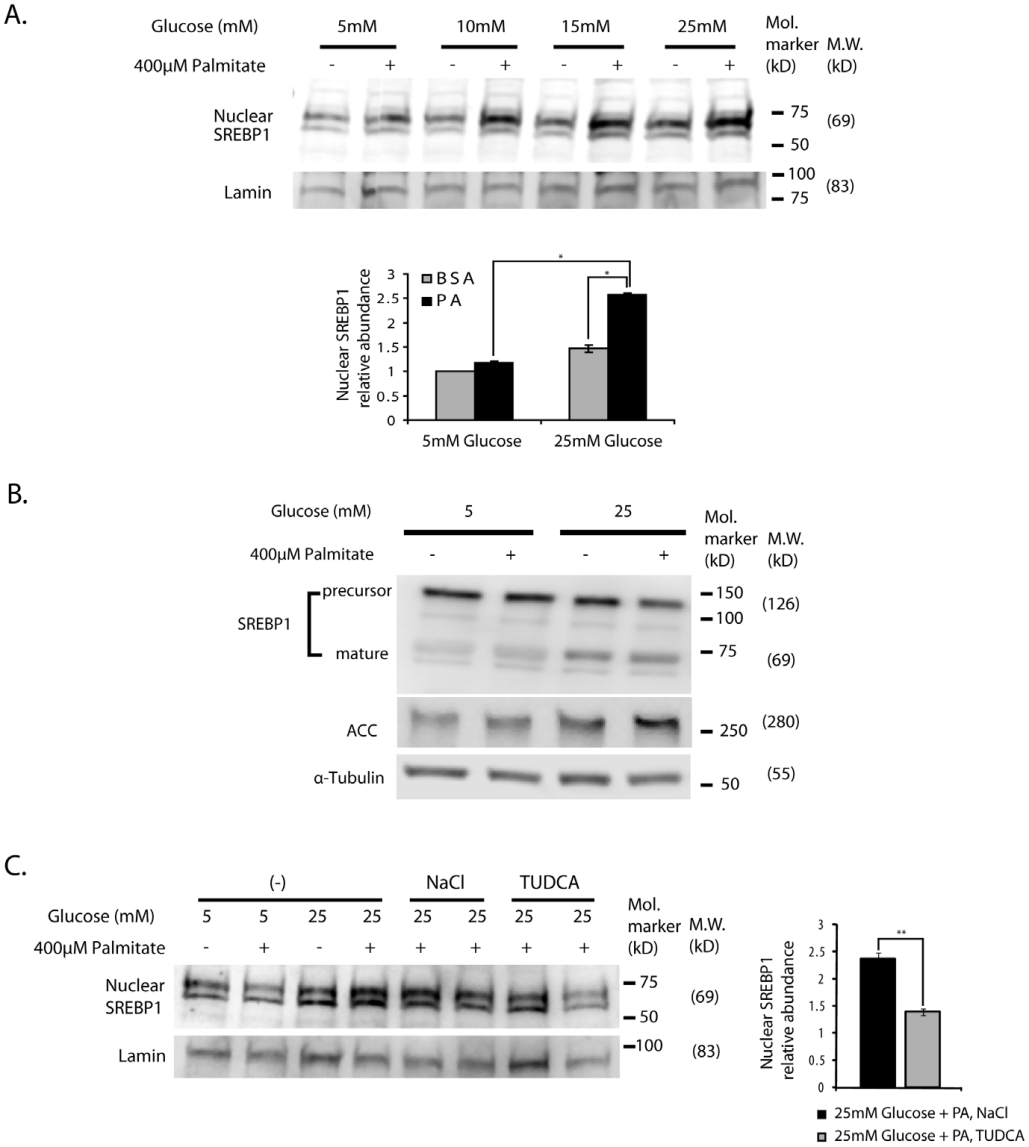
The synergistic effects of glucose and palmitate on ER stress results in concomitant effects on activation of SREBP1. (**A**) MIN6 cells were treated with either control 0.5% BSA or 400 µM palmitate+0.5% BSA at a concentration of 5, 10, 15, 25 mM glucose for 18-h. Nuclear fractions were extracted from the cells and were subjected to Western blot analyses using anti-SREBP1 and anti-Lamin antibodies. 25 µg of nuclear protein was loaded in each lane. The upper band normalized over Lamin was used to do the quantification (the lower band is nonspecific). The relative ratio of nuclear SREBP1 over Lamin calculated by densitometries was summarized as means ± S.E.M. in the graph respectively. The representative results of three experiments are shown, and graphically illustrated, * p<0.05. (**B**) MIN6 cells were treated with either control 0.5% BSA or 400 µM palmitate+0.5% BSA at a concentration of either 5 or 25 mM glucose for 24-h. Total cell lysates were subjected to Western blot analysis using anti-acetyl CoA carboxylase (ACC) and anti-α-Tubulin antibodies. The representative results of two individual experiments are shown. (**C**) Cells were treated with either 500 µg/ml NaCl or 500 µg/ml TUDCA 15-h prior to beginning of palmitate treatment. Cells were co-treated with either 0.5% BSA or 400 µM palmitate+0.5% BSA with 25 mM glucose and NaCl or TUDCA for 18-h. Nuclear fractions were extracted from the cells and were subjected to Western blot analyses using anti-SREBP1 and anti-Lamin antibodies. The upper band normalized over Lamin was used to do the quantification (the lower band is nonspecific). 25 µg of nuclear extracts were loaded in each lane. The representative results of three individual experiments are shown. The relative ratio of nuclear SREBP1 over Lamin calculated by densitometries was summarized as means ± S.E.M. in the graph respectively **p<0.01.

Sterol regulatory element-binding protein-1 (SREBP1) is a transcription factor that stimulates expression of lipid-regulatory genes [27]. SREBP1 is an ER membrane resident protein that in response to sterol depletion is cleaved to generate a transcriptionally active N-terminal fragment that translocates to the nucleus [28]. SREBP1 is increased in liver and islets of diabetic animals [29]. *In vivo* SREBP1 overexpression increased lipid accumulation in islets, reduced β-cell mass, and impaired insulin secretion [30]. Overexpression of the SREBP1 gene in insulinoma and islet β-cells also reduced IRS2 protein [31]. Furthermore, incubation of insulinoma cells and islets with high glucose (25 mM) was shown to activate SREBP1. The latter study examined only high glucose, and did not include FFA. As shown in Figure 4A, like the study of Wang et. al [31] we observed an apparent slight effect of increasing glucose on SREBP1 activation, when nuclear SREBP1 was corrected for nuclear Lamin. A synergistic effect was observed of glucose and FFA on activation of SREBP1 with a concomitant reduction of SREBP1 precursor and appearance of mature or nuclear SREBP1. SREBP1 activation was further confirmed by increased expression of its target acetyl-CoA carboxylase (ACC) (Figure 4B). SREBP1 activation was reduced by attenuation of ER stress with TUDCA pretreatment (Figure 4C) that suggested that the synergistic effects of glucose and palmitate on activation of SREBP1 were mediated by exacerbation of ER stress.

### Glucose/palmitate activate ER stress and reduce insulin signaling in primary islets

**Figure 5:**
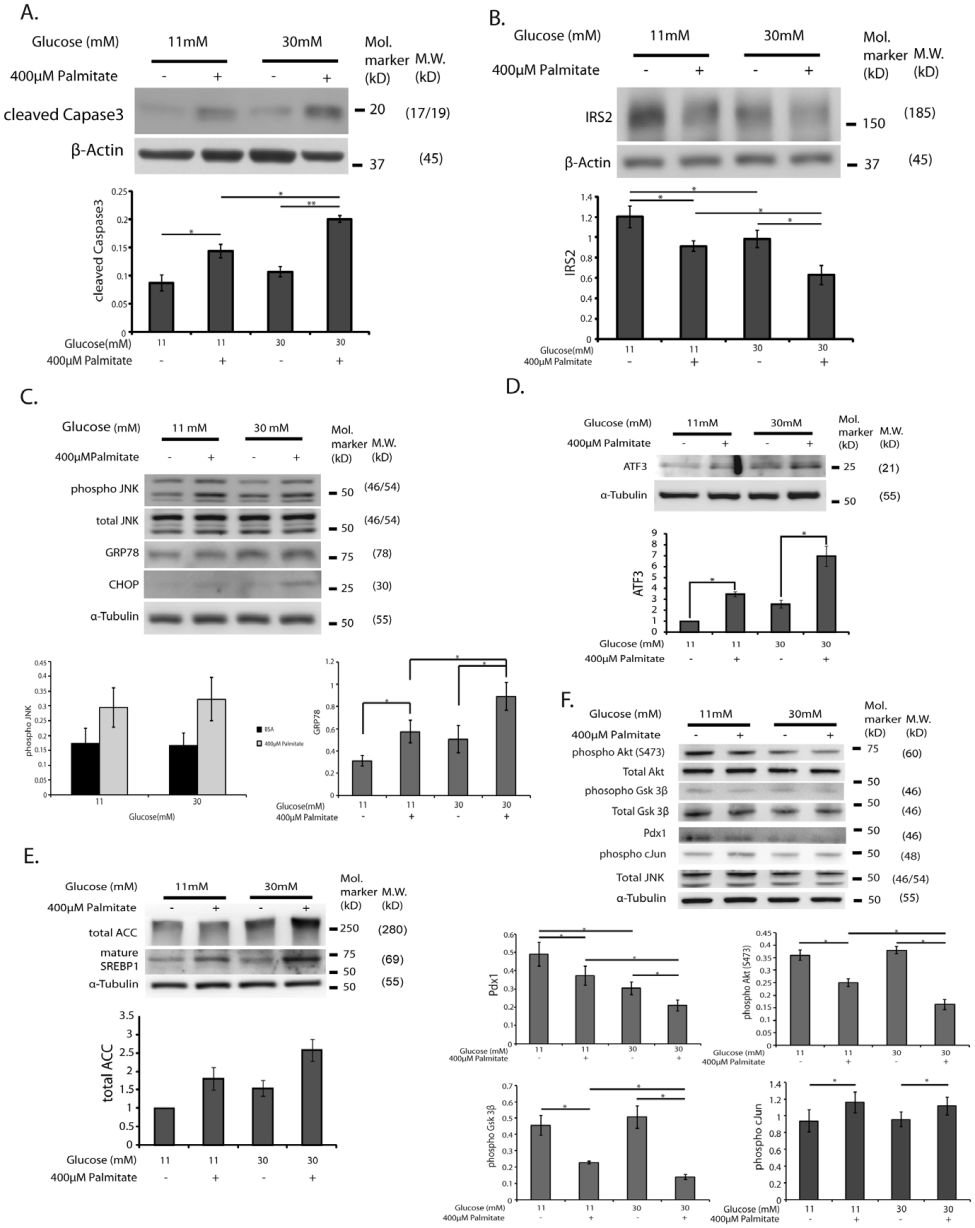
Synergistic effects of glucose and palmitate on ER stress and suppression of IRS2 expression levels in primary mouse islets. Islets from 14 weeks of age C57BL/6 male mice were isolated as described in Methods and were treated with either control 0.5% BSA or 400 µM palmitate+0.5% BSA in RPMI medium containing either 11 mM or 30 mM glucose, 10% FBS for 72-h. Total cell lysates were extracted from the islets and subjected to Western blot analysis using (**A**) anti-cleaved Caspase3 and anti-β-Actin, (**B**) anti-IRS2 antibodies, (**C**) anti-phospho-JNK, anti-total JNK, anti-GRP78, anti-CHOP, anti-α-Tubulin antibodies, (**D**) anti-ATF3 antibodies, (**E**) anti-Acetyl CoA Carboxylase (ACC), anti-SREBP1, anti-α-Tubulin antibodies, (**F**) anti-Pdx1, anti-phospho-Gsk3β, anti-total Gsk3β, anti-phospho-Akt (S473), anti-phospho-cJun, anti-α-Tubulin antibodies. The blots shown are representative of 3 individual islet experiments. The relative ratio of indicated protein over β-Actin or α-Tubulin as a loading control calculated by densitometries was summarized as means ± S.E.M. in the graph respectively *p<0.05, **p<0.01.

To validate the relevance of synergistic effects of glucose and palmitate on pancreatic β-cells we treated isolated primary mouse islets with 11 mM or 30 mM glucose in either the absence or presence of 400 µM palmitate for 72 hours. This incubation time and different glucose concentrations were utilized as it was determined that primary islets are more resistant to FFA induced apoptosis than are insulinoma cells (data not shown). Treatment with high glucose (30 mM) and palmitate resulted in an apparent but not significant (p = 0.14) enhanced induction of cleaved Caspase3 (Figure 5A), and an apparent but not significant (p = 0.07) reduced IRS2 expression (Figure 5B). In addition, high glucose and palmitate synergistically enhanced the ER stress marker GRP78 (p<0.05) beyond JNK activation (Figure 5C), and caused an apparent increase in ATF3 (p = 0.06) (Figure 5D). Consistent parallel increases in nuclear SREBP1 and total ACC protein also appeared to be potentiated by high glucose and palmitate although the effects were not significant (Figure 5E). Treatment with high glucose and palmitate did however result in significant reduction of phospho-Akt, Pdx1, and phospho-Gsk3b with no change in phospho-cJun (Figure 5F). These data together support the relevance of findings in insulinoma cells to primary islet β-cells.

### Loss-of-function of ATF3 and gain-of function of IRS2 reduce the effects of glucose and palmitate on apoptosis

To assess the effects of ER stress-induced ATF3 expression and subsequent suppression of IRS2 on glucolipotoxicity mediated apoptosis, loss- and gain-of-function studies were conducted. INS-r3 cells were utilized as the reagents for small hairpin RNA (shATF3) were controlled for rat ATF3 and not mouse. A similar role for ATF3 in MIN6 and INS1 insulinoma cell stress response has been observed (Zmuda and Hai, unpublished observations). INS-r3 cells were incubated with 400 µM palmitate/25 mM glucose for the indicated times in the presence of adenoviruses expressing either control or shATF3 to reduce ATF3 expression [14]. Note that phospho-Akt appeared to be elevated in cells expressing shATF3 at all times consistent with the known inhibitory effect of ATF3 on IRS2 transcription and subsequent insulin signaling. The IRS2 levels in the ATF3 knockdown cells appeared to be elevated relative to that in control cells at both 8 and 16 hours of glucose/palmitate treatment. Further cleaved Caspase3, following glucose/palmitate treatment, was suppressed in ATF3 knockdown cells (p<0.002). The conclusion that ER stress activates ATF3 that contributes to impaired insulin signaling and apoptosis is thus supported by these ATF3 knockdown experiments.

**Figure 6:**
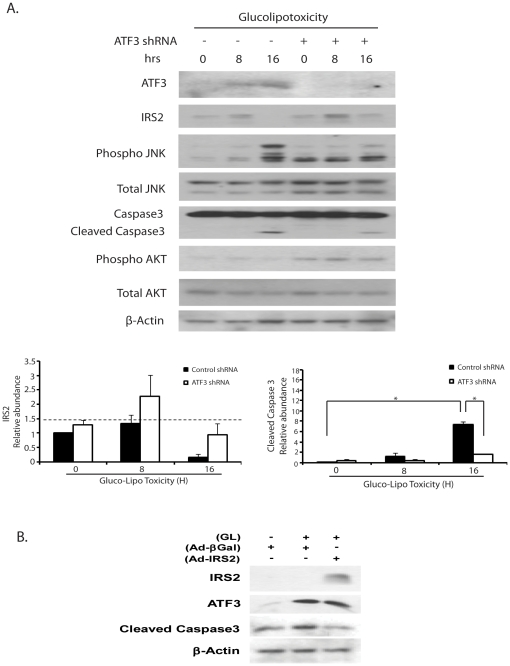
Loss-of-function of ATF3 and gain-of function of IRS2 reduce the effects of glucose and palmitate on apoptosis. (**A**) INS-r3 cells were infected with either control or ATF3 shRNA adenovirus 24-h prior to treatment with 400 µM palmitate+0.5% BSA and 25 mM glucose for the indicated times. Total cell lysates were obtained and subjected to Western blot analysis with antibodies to the indicated proteins. The relative ratio of IRS2 and cleaved Caspase3 expression over β-Actin was quantified by densitometry. The data obtained from three individual experiments are expressed as means ± S.E.M. * p<0.02, ** p<0.012. (**B**) In a single experiment, primary mouse islets were infected with adenovirus expressing βgal or IRS2 expressing adenovirus prior to treatment with either control 0.5% BSA and 5.5 mM glucose or 400 µM palmitate+0.5% BSA and 25 mM glucose for 72-h. “GL” refers to incubation in 25 mM glucose and 400 µM palmitate. Total cell lysates were subjected to Western blot using antibodies to indicated proteins.

In another experiment using primary islets treated with glucose/palmitate for 72 hours, the effects of transfection with an adenovirus expressing IRS2 was compared to that of islets with control adenovirus. As shown in Figure 6B, where Immunodetection was set to assess high levels of IRS2 in IRS2 transfected cells, there was a marked increase in IRS2 protein with IRS2 overexpression. Glucose/palmitate treatment resulted in increased ATF3 and cleaved Caspase3 with control adenovirus, while overexpression of IRS2 appeared to reduce the degree of apoptosis as measured by reduction of cleaved Caspase3 (Figure 6B). These results are consistent with the previously demonstrated role of ATF3 on ER stress induced apoptosis in islets [14], [25].

### Loss-of-function of Gsk3β on glucose and palmitate induced β-cell apoptosis

**Figure 7:**
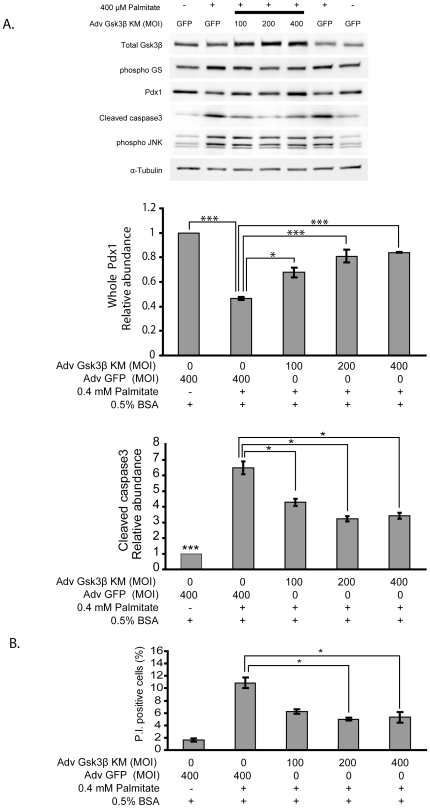
Inhibition of Gsk3β protects against glucose and palmitate-induced apoptosis in MIN6 cells. MIN6 cells were infected with 100, 200, or 400 MOI of adenovirus expressing a catalytically inactive mutant of the human Gsk3β (Adv-Gsk3βKM) or adenovirus expressing GFP (Adv-GFP) 24 hours prior to palmitate treatment. Cells were treated with 25 mM glucose and with either 0.5% BSA or 400 µM PA+0.5% BSA for 24 hours. A GFP control was placed on either end of the blot to facilitate comparison of control and Adv-Gsk3βKM. (**A**) Western blots using the indicated antibodies, with relative expression of Pdx1 normalized over α-Tubulin, and expression levels of cleaved Caspase3 normalized over α-tubulin. (**B**) Percentage of Propidium Iodide incorporation (n = 3, means ± S.E.M., *p<0.05, *** p<0.001).

The progressive decline in insulin receptor substrate signaling observed with decreasing IRS2 expression and phospho-Akt was associated with decreased phosphorylation of Gsk3β (Figure 2) and thus activation of the pro-apoptotic form of Gsk3β [32]. To examine the contribution of activation of Gsk3β, the effects of palmitate treatment with increasing amounts of an adenovirus expressing a catalytic inactive mutant of the human Gsk3β (Adv-Gsk-3βKM) were analyzed by Western blot (Figure 7A). A control sample was placed on either end of the blot to facilitate comparisons. For instance, observe the increase in cleaved Caspase3 and phopho-JNK between the two controls on either end of the blot. Increasing doses of the virus correlated with increased levels of total Gsk-3β, and reduced levels of the Gsk-3β substrate phospho-GS. Increased kinase dead Gsk-3β virus also resulted in increased expression of Pdx1, reduced apoptosis as suggested by cleaved Caspase 3 levels, and cell death measured by propidium iodide incorporation (Figure 7B). Interestingly the protective effects of the Adv-Gsk-3βKM occurred in spite of apparent comparable activation of phospho-JNK with palmitate treatment.

## Discussion

The combination of hyperglycemia and hyperlipidemia that is associated with insulin resistance may contribute to reducing β-cell mass and promoting the transition to full blown Type 2 diabetes, but the underlying mechanisms are only partially understood. This study examined the sequence of molecular events that may be involved in this process, and resulted in several novel observations: 1) While early induction of JNK plays a role in FFA-induced apoptosis [13], [16], it does not appear involved in the high glucose potentiation of FFA effects; 2) the potentiating effects of glucose and FFA on ER stress result in activation of ER-associated SREBP1 and ATF3 leading to reduced IRS2 expression further impairing insulin-receptor substrate-PI-3K-Akt signaling, and 3) treatment with an adenovirus expressing a kinase dead Gsk3β significantly restored Pdx1 levels, and reduced the apoptosis induced by high glucose and FFA. Together these findings provide a molecular model for the synergistic effects of glucose and FFA on islet cell death and identify potentially useful therapeutic targets.

The role of JNK activation in FFA-induction of apoptosis in β-cells has been documented [13], [16], [33]. In the current study, JNK activation by palmitate was significant as expected, but was maximal at low glucose, and glucose potentiation of FFA-induced apoptosis was JNK independent (Figure 1B). How FFA treatment of β-cells leads to JNK activation is not completely known, but FFA induce ER stress, which activates JNK activation, and JNK activation itself can induce ER stress [13], [34]. Interestingly, TUDCA appeared to reduce cleaved Caspase3 and CHOP induction, while not appearing to reduce JNK activation (Figure 3) suggesting that in this case JNK activation is upstream of ER stress. On the other hand the results of inhibition of ATF3 by shRNA in Figure 6A suggest that JNK activation is upstream of ER stress. Our results do not therefore settle this issue of the relative position of JNK activation and ER stress. Regardless of whether JNK activation is upstream or downstream of ER stress, the findings in the current study show that the combination of high glucose and FFA does not associate with further activation of JNK as compared to FFA in low glucose. This result is to some extent in conflict with that of Bachar et. al. [13] who showed that incubating islet cells in low vs. high glucose resulted in both increased ER stress and JNK activation. A difference in the two experiments is that Bachar et.al. evaluated islet cells in 3.3 mM vs. 22 mM glucose, and our experiments assessed cells in 5.5 mM vs. 25 mM glucose. Our results do not rule out the contributory effect of JNK on β-cell apoptosis as previously shown [13], [16], [33] but they emphasize the importance of additional mechanisms contributed by high glucose. Additionally, since glucose and palmitate have been shown to evoke oxidative stress, impairing nuclear translocation of Pdx1 and triggering β-cell failure [35], it is conceivable that oxidative stress interacts with JNK, ATF3 and ER stress to contribute to glucose/palmitate induced apoptosis, although this hypothesis remains to be tested.

The role of SREBP1 in high glucose induced apoptosis in islet β-cells has been reported [31]. In the absence of FFA, high glucose alone for 48 hours was shown to activate SREBP1 and to repress IRS2 and Pdx1 levels. Expression of a dominant negative SREBP1 reversed these transcriptional effects. In the current study, glucose alone at high concentration slightly activated SREBP1 as shown in Figure 4, an event that did not correlate with ER stress or apoptosis. A much more significant activation of SREBP1 was observed with glucose and palmitate together and this was shown to be a function of induced ER stress since it was attenuated by TUDCA (Figure 4C). These results only show an association of SREBP1 nuclear translocation, and do not document its causal role. It is likely however that SREBP1 nuclear translocation participates in glucose/palmitate induced apoptosis, as previous studies documented the causal role of SREBP1 in ER stress induced apoptosis in insulinoma cells [31]. SREBP1 resides in the ER membrane, where it is anchored by the labile protein INSIG1 [36]. The link between ER stress and SREBP1 activation has been little studied. Lee et. al. using CHO cells [36] showed that thapsigargin, a chemical that induces ER stress, activates SREBP1 due to rapid degradation of INSIG1 [36]. We observed that glucose and palmitate together appeared to reduce INSIG1 protein (data not shown), which likely contributed to augmentation of nuclear SREBP1 under these conditions.

Similarly a synergistic effect of glucose and FFA was observed on expression of the ER-associated stress marker ATF3. ATF3 expression paralleled suppression of IRS2 protein levels, and induction of apoptosis measured by CHOP (Figure 3A–B), and Caspase3 activation (Figure 3A). The association of ATF3 with ER stress and cell death has been well documented [37] but there is relatively little known related to ATF3 targets [14]. Recently, ATF3 was shown to suppress the IRS2 protein by binding to the IRS2 promoter [14] and implicated this mechanism in apoptosis induced by agents such as γ-interferon, TNF-α, or thapsigargin. Our findings are consistent with the involvement of ATF3 in the apoptotic effects of nutrient induced ER stress in islet cells. In this context, we note that Cunha et. al. [33] did not find a pro-apoptotic role of ATF3 in the context of palmitate treatment. Potential explanations for this apparent difference include the difference in glucose concentration (25 mM glucose in our study and 11 mM glucose in theirs), the cells used (INS-r3 in our study and INS-1E in theirs), and assays (activated Caspase3 in our study and propidium iodide plus Hoechst stain). An interesting question is whether ATF3 is a direct repressor of Pdx1 expression. Insulin signaling alters Gsk3β and FoxO activity [16], [32], [38], [39] and these proteins are known regulators of Pdx1 expression. As ATF3 represses IRS2 and insulin signaling, at least part of ER stress and ATF3 induced Pdx1 suppression is due to decreased insulin signaling, and perhaps also due to direct suppression of Pdx1 expression, but this latter question remains to be determined by future experiments. Interestingly ATF3 induction is dependent on the P38 kinase pathway [15] which is part of signaling transduced by the membrane fatty acid translocase CD36 and a role of CD36, which is induced by glucose, has been proposed in mediating palmitate induced apoptosis of kidney tubular epithelial cell [40]. The role of this pathway in islet cells will need to be explored in future studies.

In the current studies we have shown a correlation among suppression of IRS2 protein levels (Figure 2), ATF3 expression (Figure 3), and resultant induction of apoptosis. The causal relationships among these events were demonstrated by Li. et. al. [14] when insulinoma cells and/or mouse islets were transfected with adenoviruses expressing inducible gain- or loss-of-function of ATF3 and IRS2. Treatments that induced ATF3 activation and IRS2 suppression included induction of apoptosis by combined treatment of insulinoma cells with γ-interferon, TNF-α, or the ER stress activator thapsigargin. These studies demonstrated that ATF3, like the transcription factor CREB, alters IRS2 expression by binding to the IRS2 promoter. In the current study, we have examined the role of combined glucose and palmitate on this pathway (Figure 6). Transduction with an adenovirus expressing shATF3 significantly reduced this effect, while transduction with AdV-IRS2 ameliorated the apoptotic effect, thus mechanistically linking this pathway.

In this study co-incubation of insulinoma cells with an adenovirus expressing a kinase dead Gsk3β (Adv-Gsk3βKM, Figure 7) along with high glucose and palmitate for 24 hours significantly reduced cleaved Caspase3 and cell death. A previous study in IRS2 null mice had demonstrated that the severe diabetes associated with markedly increased apoptosis and reduced proliferation of islet β-cells was reversed when crossed with Gsk3β haploinsufficient mice [32]. The double knockout was also associated with enhanced expression of Pdx1 in islet β-cells. Like the IRS2 null mice with severe insulin resistance, glucose and FFA treatment of insulinoma cells and primary islets induces a state of insulin resistance. Also like IRS2 null mice on the Gsk3β genetic deficient background, reducing Gsk3β activity with a kinase dead Gsk3β adenovirus restored Pdx1 levels and reduced apoptosis and cell death. These findings emphasize the contribution of impaired insulin receptor substrate signaling in the apoptosis of β-cells treated with glucose and FFA, and the contribution of Gsk3β activity to this process.

**Figure 8:**
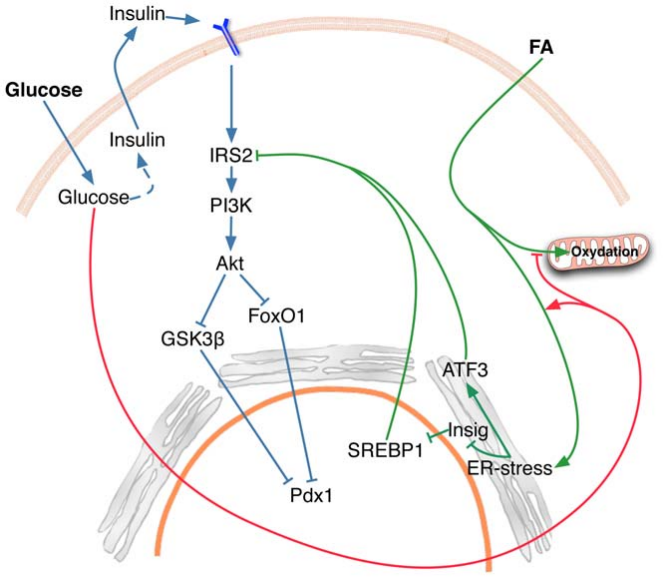
Working diagram illustrating some of the key steps involved in “glucolipotoxicity” of β-cells. High glucose and FFA together result in a vicious negative cycle that ultimately promotes β-cell death. As suggested by our findings, high glucose addition to FFA treated β-cells results in much more activation of SREBP1 than glucose alone. SREBP1 enhances ACC expression with generation of malonyl-CoA which impairs FFA oxidation. This in turn leads to augmented ER stress with further activation of ER-localized SREBP1 as a result of degradation of the anchoring protein Insig1. The excess non-metabolized FFA due to more impairment of FFA oxidation would partition in ER membranes compounding ER stress. In addition to SREBP1, ER stress activates ATF3. Both nuclear SREBP1 and ATF3 result in inhibition of IRS2, with concomitant impairment of insulin signaling, activation of Gsk3β and reduction of Pdx1 leading to apoptosis.

A schematic diagram highlights the key concepts implied by our findings (Figure 8). In the insulin resistant subject abnormal metabolism of FFA and glucose result in chronically high levels of both nutrients in the circulation [41]. Under such conditions, the combination of hyperglycemia and FFA has been suggested to be particularly harmful for β-cells leading to so-called glucolipotoxicity [6], [42], [43]. Our findings suggest that addition of high glucose to FFA treated β-cells results in an escalating negative cycle. Under such conditions activation of the transcription factor SREBP1 leads to enhanced ACC expression with generation of malonyl-CoA (MCC), which impairs FFA oxidation. These in turn lead to ER stress with further activation of SREBP1, ATF3, and impairment of FFA oxidation. The excess unmetabolized FFA would partition in ER membranes compounding ER stress. Additionally nuclear SREBP1 and ATF3 result in inhibition of IRS2, with concomitant impairment of insulin receptor substrate signaling, increase of Gsk3β activity and reduction of Pdx1 leading to apoptosis. Several steps in the cycle shown in Figure 8 may be amenable to therapeutic intervention. These include the impairment of FFA oxidation by glucose, the synergistic effects of glucose and FFA on ER stress, the activation of SREBP1 and its negative effects on insulin signaling, and the downstream mediators of apoptosis that are activated by reduced IRS-signaling involving both Gsk-3β and Fox01.

The results of the current studies illustrate some of the potential mechanisms whereby a combination of high glucose and FFA, as occurs in insulin resistant subjects, may result in eventual destruction of β-cells. However, the ultimate contribution of these mechanisms to the etiology of β-cell failure and diabetes remains unknown and will need to be validated *in vivo*.
